# Supplementary figures and images for: Differentiation patterns of emperor moths (Lepidoptera: Saturniidae: Saturniinae) of a continental island: divergent evolutionary history driven by Pleistocene glaciations
Source: PeerJ. 2022 Apr 18;10:e13240. doi: 10.7717/peerj.13240 (PMC9022646; doi:10.7717/peerj.13240)

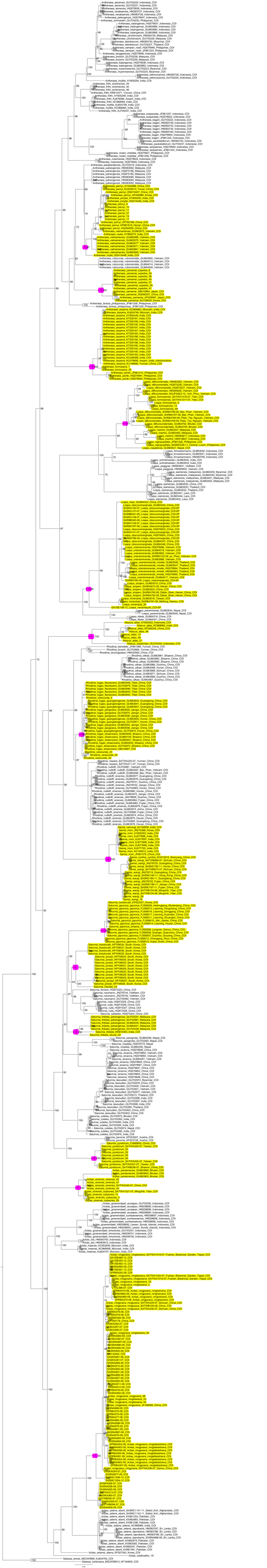

Supplement: Supplemental Information 2 — The COI sequences of 519 individuals across 114 saturniid species related to Formosan genera of Actias, Antheraea, Attacus, Loepa, Rhodinia, Samia, and Saturnia were included. Bootstrap values of the ML tree are provided beneath the node. [file peerj-10-13240-s002.pdf]

## *Rhodinia verecunda*

(A)

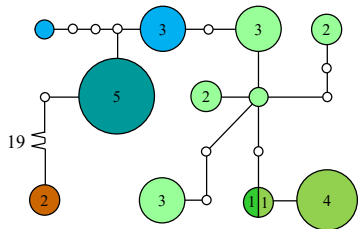

**(B)**

|       |       |       |       |
|-------|-------|-------|-------|
|       | Rho_v | Rho_f | Rho_n |
| Rho_v | 0.3   | 0.7   | 3.7   |

(C)

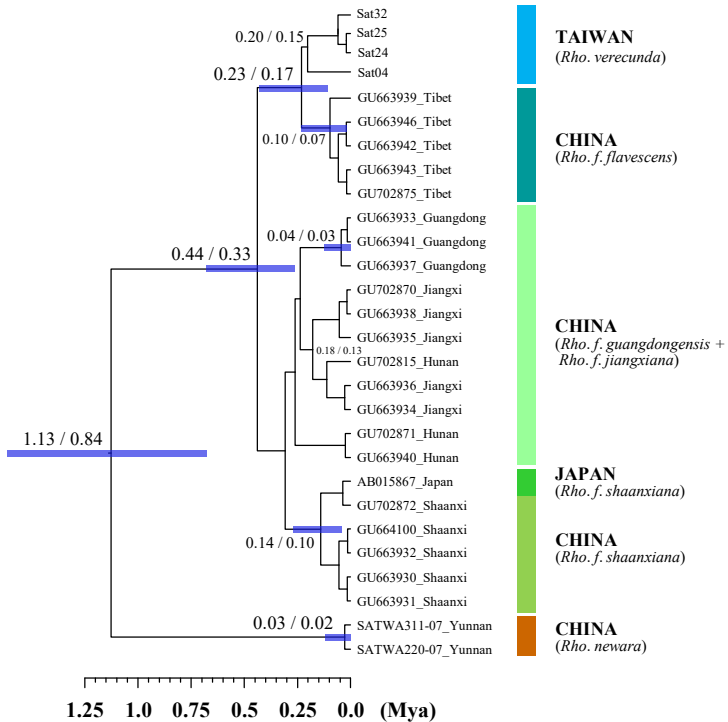

Supplement: Supplemental Information 3 — The samples and geographical distributions of all Rhodinia species are indicated by different colors. The abbreviations Rho_v, Rho_f, and Rho_n represent Rho.verecunda, Rho. fugax, and Rho. newara, respectively. In the haplotype network (A), each circle represents a haplotype connected to another haplotype through one substitution step, and the numbers of substitution steps greater than one are marked. The number of haplotype individuals greater than one is marked inside the circle, with the smallest circle corresponding to one individual. Sequence divergences (B) in pairwise taxa are provided as percentages (%). Calibration dating is provided in millions of years ago (Mya) (C). [file peerj-10-13240-s003.pdf]

*Loepa formosensis*

(A)

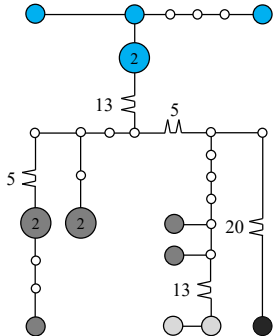

(B)

|       | Loe_f | Loe_d_V | Loe_d_B | Loe_n |
|-------|-------|---------|---------|-------|
| Loe_f | 0.4   | 3.5     | 4.7     | 5.3   |

(C)

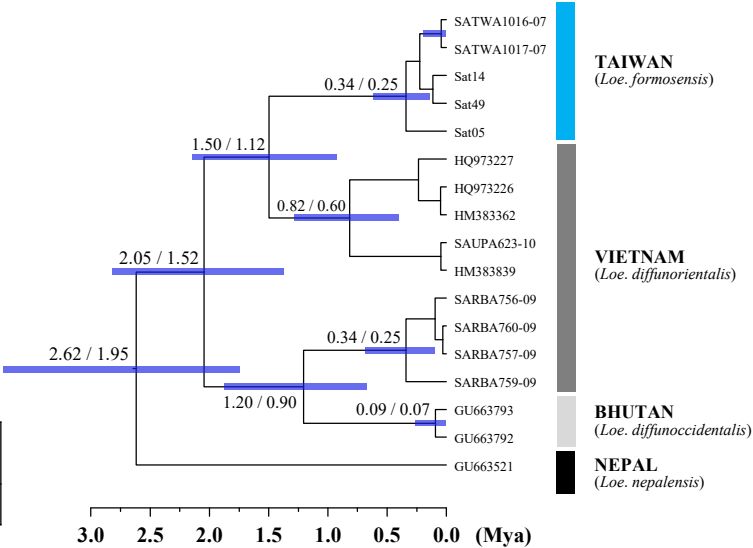

Supplement: Supplemental Information 4 — The samples and geographical distributions of all Loepa species are indicated by different colors. The abbreviations Loe_f, Loe_d_V, Loe_v_B, and Loe_n refer to Loe. formosensis, Loe. diffunorientalis_Vietnam, Loe. diffunoccidentalis_Bhutan, and Loe. nepalensis, respectively. In the haplotype network (A), each circle represents a haplotype connected to another haplotype through one substitution step, and the numbers of substitution steps greater than one are marked. The number of haplotype individuals greater than one is marked inside the circle, with the smallest circle corresponding to one individual. (B) Sequence divergences in pairwise taxa are provided as percentages (%). (C) Calibration dating is provided in millions of years ago (Mya) (C). [file peerj-10-13240-s004.pdf]

# *Antheraea superba*

(A)

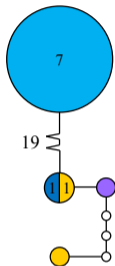

(B)

|       | Ant_s | Ant_y |
|-------|-------|-------|
| Ant_s | 0     | 3.8   |

(C)

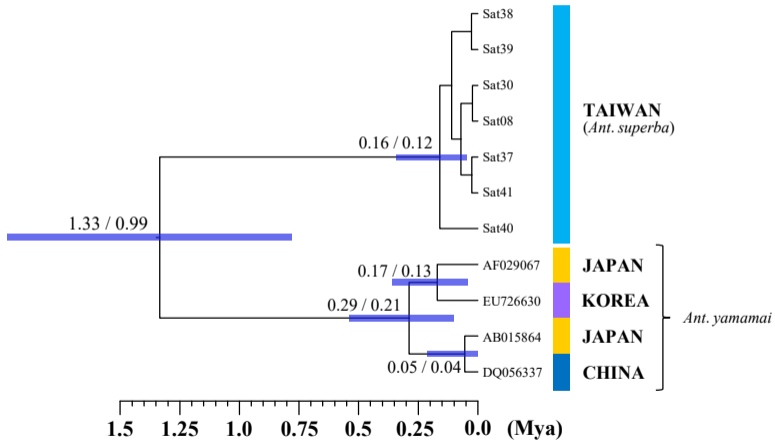

Supplement: Supplemental Information 5 — The samples and geographical distributions of Ant_s and Ant_y are indicated by different colors. In the haplotype network (A), each circle represents a haplotype connected to another haplotype through one substitution step, and the numbers of substitution steps greater than one are marked. The number of haplotype individuals greater than one is marked inside the circle, with the smallest circle corresponding to one individual. Sequence divergences (B) in pairwise taxa are presented as percentages (%). Calibration dating is provided in millions of years ago (Mya) (C). [file peerj-10-13240-s005.pdf]

# *Antheraea formosana*

(A)

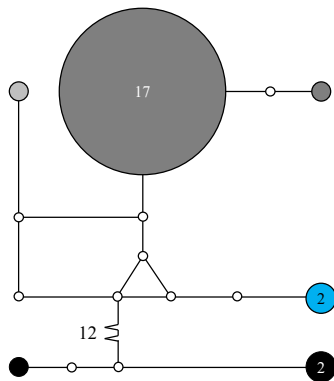

(B)

|       | Ant_f | Ant_a | Ant_p |
|-------|-------|-------|-------|
| Ant_f | 0.4   | 1.1   | 3.4   |

(C)

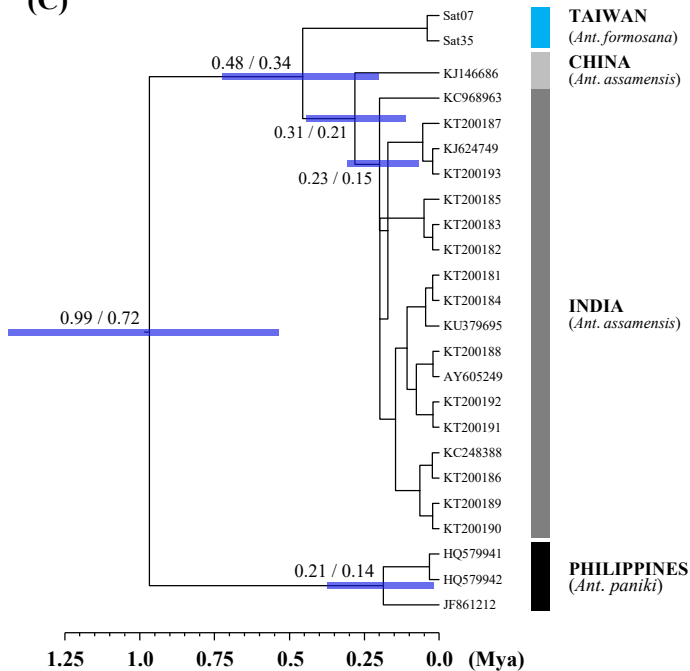

Supplement: Supplemental Information 6 — The samples and geographical distributions of all Antheraea species are indicated by different colors. The abbreviations Ant_f, Ant_a, and Ant_p refer to Ant. formosana, Ant. assamensis, and Ant. paniki, respectively. In the haplotype network (A), each circle represents a haplotype connected to another haplotype through one substitution step, and the numbers of substitution steps greater than one are marked. The number of haplotype individuals greater than one is marked inside the circle, with the smallest circle corresponding to one individual. (B) Sequence divergences in pairwise taxa are provided as percentages (%). (C) Calibration dating is provided in millions of years ago (Mya). [file peerj-10-13240-s006.pdf]

# *Saturnia fukudai*

(A)

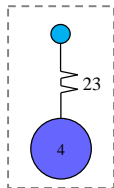

(B)

|       | Sat_f | Sat_b | Sat_j |
|-------|-------|-------|-------|
| Sat_f | -     | 3.6   | 4.3   |

(C)

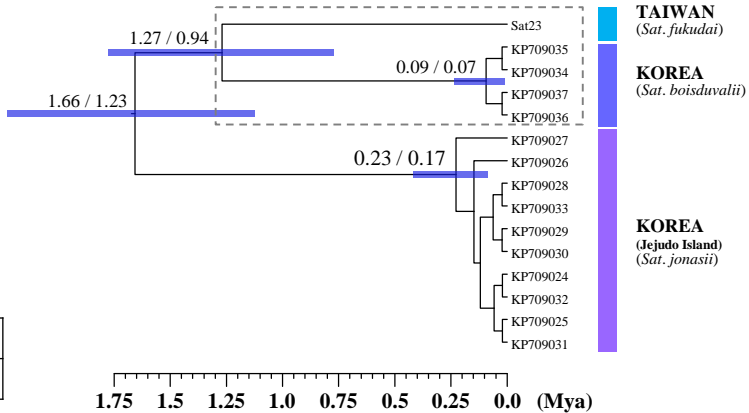

Supplement: Supplemental Information 7 — The samples and geographical distributions of all Saturnia species are indicated by different colors. The abbreviations Sat_f, Sat_b, and Sat_j refer to Sat. fukudai, Sat. boisduvalii, and Sat. jonasii, respectively. In the haplotype network (A), each circle represents a haplotype connected to another haplotype through one substitution step, and the numbers of substitution steps greater than one are marked. The number of haplotype individuals greater than one is marked inside the circle, with the smallest circle corresponding to one individual. Sequence divergences (B) in pairwise taxa are presented as percentages (%). Calibration dating is provided in millions of years ago (Mya) (C). [file peerj-10-13240-s007.pdf]

# *Saturnia thibeta okurai*

(A)

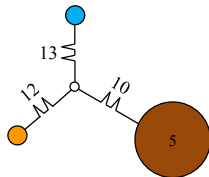

(B)

|           | Sat_thi_o | Sat_thi_p | Sat_thi_e |
|-----------|-----------|-----------|-----------|
| Sat_thi_o | -         | 3.9       | 4.2       |

(C)

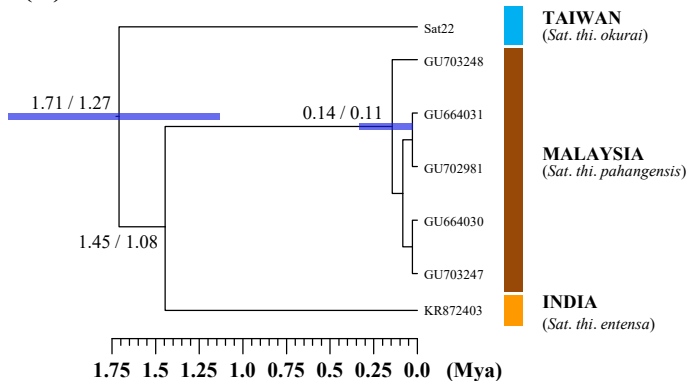

Supplement: Supplemental Information 8 — The samples and geographical distributions of all Sat. thibeta subspecies are indicated by different colors. The abbreviations Sat_t_o, Sat_t_p, and Sat_t_e represent Sat. thi. okurai, Sat. thi. pathangensis, and Sat. thi. extensa, respectively. In the haplotype network (A), each circle represents a haplotype connected to another haplotype through one substitution step, and the numbers of substitution steps greater than one are marked. The number of haplotype individuals greater than one is marked inside the circle, with the smallest circle corresponding to one individual. Sequence divergences (B) in pairwise taxa are presented as percentages (%). Calibration dating is provided in millions of years ago (Mya) (C). [file peerj-10-13240-s008.pdf]

*Saturnia japonica arisana*

(A)

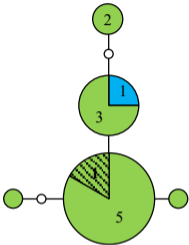

(B)

|           | Sat_jap_a | Sat_jap_j |
|-----------|-----------|-----------|
| Sat_jap_a | -         | 0.2       |

(C)

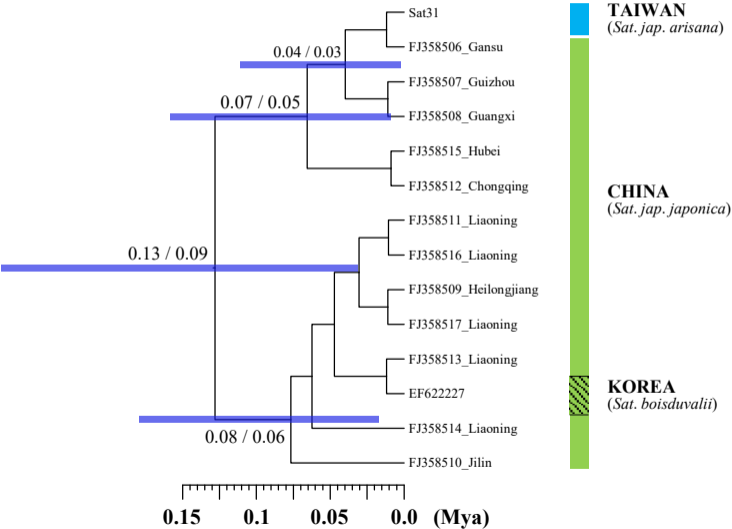

Supplement: Supplemental Information 9 — The samples and geographical distributions of all Sat. japonica subspecies are indicated by different colors. The abbreviations Sat_jap_a and Sat_jap_j refer to Sat. jap. arisana and Sat. jap. japonica, respectively. In the haplotype network (A), each circle represents a haplotype connected to another haplotype through one substitution step, and the numbers of substitution steps greater than one are marked. The number of haplotype individuals greater than one is marked inside the circle, with the smallest circle corresponding to one individual. Sequence divergences (B) in pairwise taxa are presented as percentages (%). Calibration dating is provided in millions of years ago (Mya) (C). [file peerj-10-13240-s009.pdf]

*Actias sinensis subaurea*

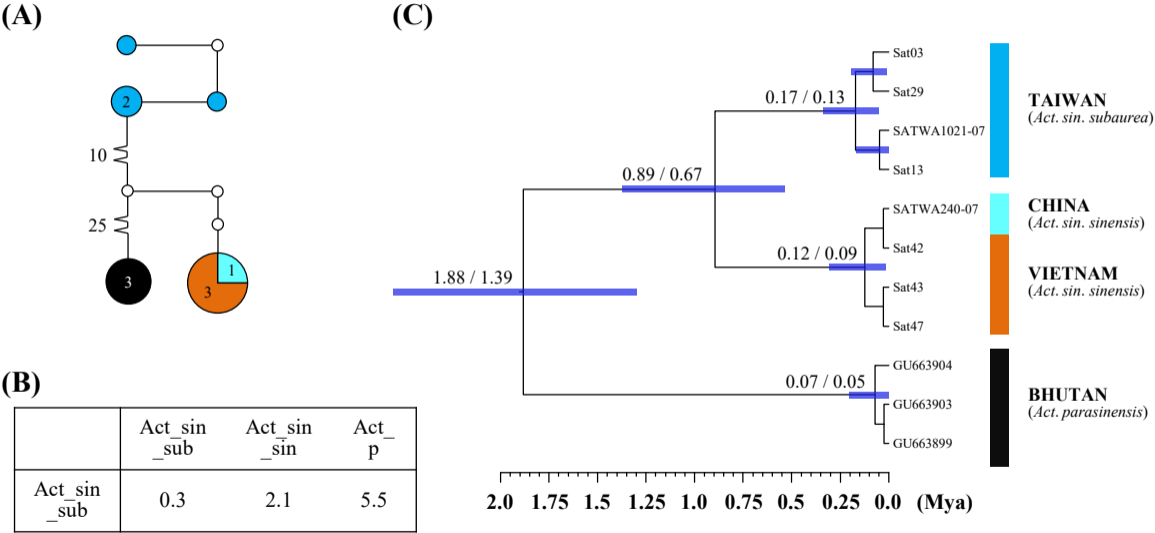

Supplement: Supplemental Information 10 — The samples and geographical distributions of all Actias species are indicated by different colors. The abbreviations Act_s_sub, Act_s_sin, and Act_p refer to Act. sin. subaurea, Act. sin. sinensis, and Act. parasinensis, respectively. In the haplotype network (A), each circle represents a haplotype connected to another haplotype through one substitution step, and the numbers of substitution steps greater than one are marked. The number of haplotype individuals greater than one is marked inside the circle, with the smallest circle corresponding to one individual. Sequence divergences (B) in pairwise taxa are presented as percentages (%). Calibration dating is provided in millions of years ago (Mya) (C). [file peerj-10-13240-s010.pdf]

## *Antheraea pernyi*

(A)

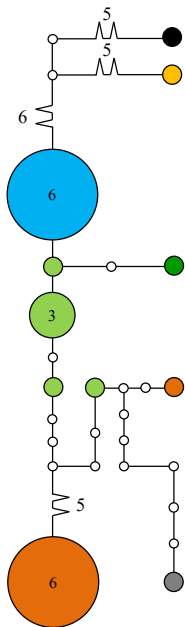

**(B)**

|       |       |       |       |       |
|-------|-------|-------|-------|-------|
|       | Ant_p | Ant_v | Ant_j | Ant_r |
| Ant_p | 0.3   | 1.7   | 2.4   | 2.6   |

(C)

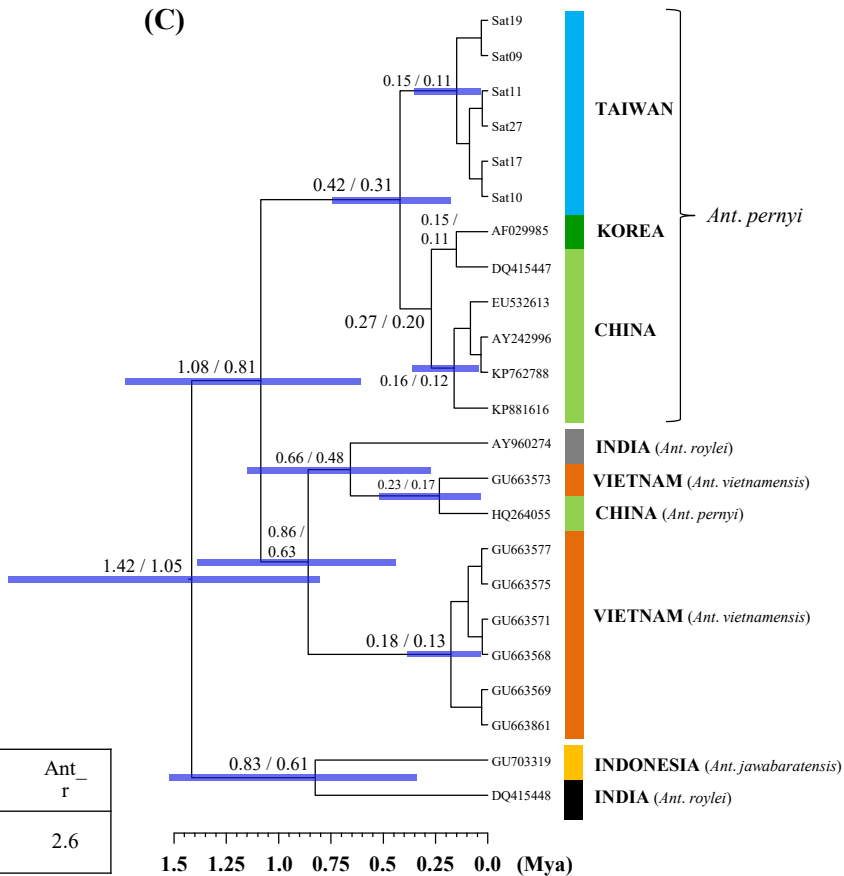

Supplement: Supplemental Information 11 — The samples and geographical distributions of all Antheraea species are indicated by different colors. The abbreviations Ant_p, Ant_v, Ant_j, and Ant_r refer to Ant. pernyi, Ant. vietnamensis, Ant. jawabaratensis, and Ant. roylei, respectively. In the haplotype network (A), each circle represents a haplotype connected to another haplotype through one substitution step, and the numbers of substitution steps greater than one are marked. The number of haplotype individuals greater than one is marked inside the circle, with the smallest circle corresponding to one individual. Sequence divergences (B) in pairwise taxa are presented as percentages (%). Calibration dating is provided in millions of years ago (Mya). [file peerj-10-13240-s011.pdf]

# *Saturnia pyretorum*

(A)

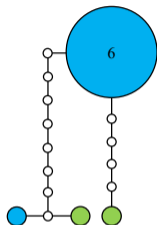

(B)

|      | T_I | T_II | C_I |
|------|-----|------|-----|
| T_II | 1.4 |      |     |
| C_I  | 0.8 | 2.2  |     |
| C_II | 1.4 | 0.3  | 2.2 |

(C)

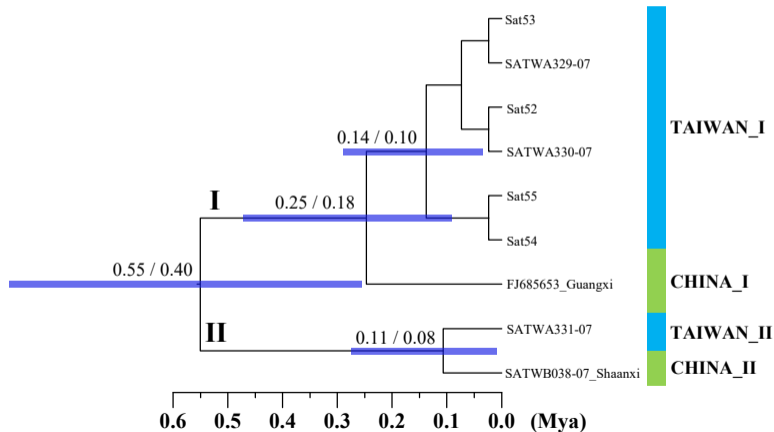

Supplement: Supplemental Information 12 — Samples from Taiwan (T) and China (C) are indicated by different colors. Lineages I and II in Sat. pyretorum are presented. In the haplotype network (A), each circle represents a haplotype connected to another haplotype through one substitution step, and the numbers of substitution steps greater than one are marked. The number of haplotype individuals greater than one is marked inside the circle, with the smallest circle corresponding to one individual. Sequence divergences (B) in pairwise taxa are presented as percentages (%). Calibration dating is provided in millions of years ago (Mya) (C). [file peerj-10-13240-s012.pdf]

# *Samia wangi*

(A)

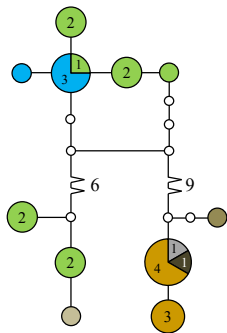

(B)

|         | Sam_w_T | Sam_w_C | Sam_cyn | Sam_r | Sam_can |
|---------|---------|---------|---------|-------|---------|
| Sam_w_T |         |         |         |       |         |
| Sam_w_C | 1.1     |         |         |       |         |
| Sam_cyn | 2.6     | 1.9     |         |       |         |
| Sam_r   | 3.4     | 3.8     | 4.6     |       |         |
| Sam_can | 3.3     | 3.7     | 4.5     | 0.2   |         |

(C)

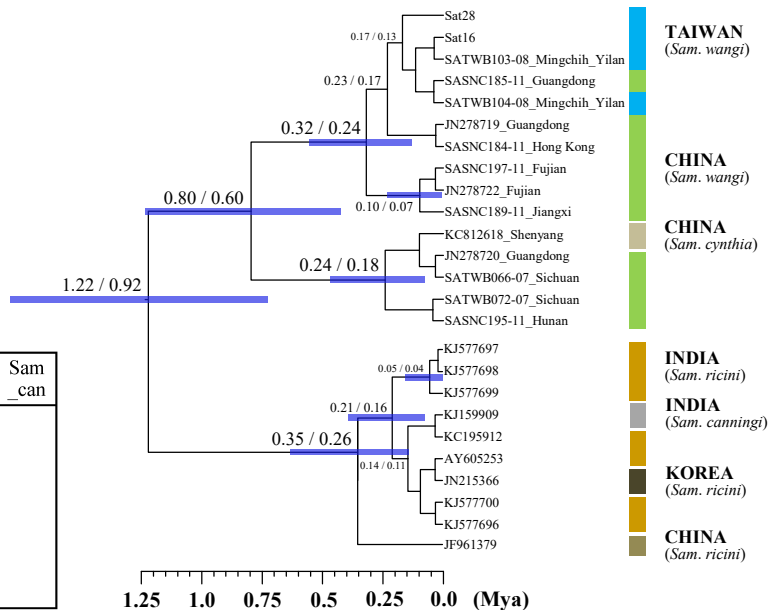

Supplement: Supplemental Information 13 — The samples and geographical distributions of Sam. wangi populations and the ally Samia species are indicated by different colors. The abbreviations Sam_w_T, Sam_w_C, Sam_cyn, Sam_r, and Sam_can refer to Sam. wangi_Taiwan, Sam. wangi_China, Sam. cynthia, Sam. ricini, and Sam. canningi, respectively. In the haplotype network (A), each circle represents a haplotype connected to another haplotype through one substitution step, and the numbers of substitution steps greater than one are marked. The number of haplotype individuals greater than one is marked inside the circle, with the smallest circle corresponding to one individual. Sequence divergences (B) in the pairwise taxa are presented as percentages (%). Calibration dating is provided in millions of years ago (Mya) (C). [file peerj-10-13240-s013.pdf]
